# Meta-analyses of gene methylation and smoking behavior in non-small cell lung cancer patients

**DOI:** 10.1038/srep08897

**Published:** 2015-03-10

**Authors:** Tao Huang, Xiaoying Chen, Qingxiao Hong, Zaichun Deng, Hongying Ma, Yanfei Xin, Yong Fang, Huadan Ye, Rujie Wang, Cheng Zhang, Meng Ye, Shiwei Duan

**Affiliations:** 1 Zhejiang Provincial Key Laboratory of Pathophysiology, School of Medicine, Ningbo University, Ningbo, Zhejiang 315211, China; 2 The Affiliated Hospital of Ningbo University, Ningbo, Zhejiang 315020, China; 3 State Key Laboratory of Safety Evaluation for New Drugs, Zhejiang Academy of Medical Sciences, Hangzhou, Zhejiang, China; 4 Department of Medical Oncology, Sir Run Run Shaw Hospital, Zhejiang University School of Medicine, Hangzhou, Zhejiang 310016, China

## Abstract

Aberrant DNA methylation can be a potential genetic mechanism in non-small cell lung cancer (NSCLC). However, inconsistent findings existed among the recent association studies between cigarette smoking and gene methylation in lung cancer. The purpose of our meta-analysis was to evaluate the role of gene methylation in the smoking behavior of NSCLC patients. A total of 116 genes were obtained from 97 eligible publications in the current meta-analyses. Our results showed that 7 hypermethylated genes (including *CDKN2A*, *RASSF1*, *MGMT*, *RARB*, *DAPK*, *WIF1* and *FHIT*) were significantly associated with the smoking behavior in NSCLC patients. The further population-based subgroup meta-analyses showed that the *CDKN2A* hypermethylation was significantly associated with cigarette smoking in Japanese, Chinese and Americans. In contrast, a significant association of *RARB* hypermethylation and smoking behavior was only detected in Chinese but not in Japanese. The genes with altered DNA methylation were likely to be potentially useful biomarkers in the early diagnosis of NSCLC.

Non-small-cell lung carcinoma (NSCLC) is a major type of epithelial lung cancer causing a serious hazard to human health^1^,^2^. The mortality of lung cancer was higher than that of any other malignant tumors among men and women in China^3^, and the incidence of lung cancer has been rising year by year^4^. Early detection is the key method to improve the survival of lung cancer patients^5^.

Cigarette smoking is the top risk factor which is attributed to more than four out of five cases of lung cancers^6^. Up to date, a handful of candidate genes have been investigated the association with cigarette smoking^7^,^8^. Epigenetic inhibition of gene expression is a potential cause for initiation and progression of lung cancer^8^. DNA methylation, a reversible epigenetic modification, plays an important role in gene expression at the very early stage of NSCLC^9^. Identification of aberrantly-methylated smoke-related genes may have potential in the early diagnosis of NSCLC.

Aberrant promoter methylation of smoking related genes has been reported in lung cancer patients^7^, but an unanimous conclusion could not be reached even in the same ethnic population^10^,^11^. Although several meta-analyses have reported the results of individual genes in patients with NSCLC, most of the current methylation studies were involved with a small number of samples that might produce spurious results. The purpose of our study was to perform an overview of all the candidate genes rather than single gene associated with smoking in NSCLC patients. Our meta-analysis would help identify the genes with robust findings across the studies in different ethnic populations.

## Methods

### Selection criteria of eligible studies

We searched the available studies through October 27, 2014 in the PubMed (English), CNKI and Wanfang (Chinese) literature databases using the corresponding combination: “(smok* OR nonsmok* OR clinic*) AND lung cancer AND (methylation OR epigenetic silencing)”. The inclusion criteria for the studies involved in this meta-analysis met the following criteria: 1) they were conducted in non-small cell lung cancer patients; 2) the subjects in every study comprised nonsmokers and smokers (former smokers and/or current smokers); 3) odds ratio (OR) with 95% confidential interval (CI) was included.

### Data Extraction

All the datasets were extracted independently by two reviewers using a standard protocol. For each eligible study, we collected information regarding the names of first authors, publication year, ethnicity, country of origin, histology of lung cancer, types of biological specimen, number of participants, methylation status, smoking status, the method for methylation detection, and OR with 95% CI.

### Meta-analysis

The meta-analysis was performed using the Review Manager software (version 5.2, Cochrane Collaboration, Oxford, United Kingdom)^12^. The combined ORs and the corresponding 95% CIs were calculated and demonstrated in the forest plots using the fixed or the random effects model. A fixed-effect model was applied for the meta-analysis with moderate heterogeneity (I^2^ < 50%), otherwise a random-effect model was used^13^. Funnel plots were used to check whether there was obvious publication bias among the involved studies. P values less than 0.05 were considered to be significant.

## Results

**Figure 1:**
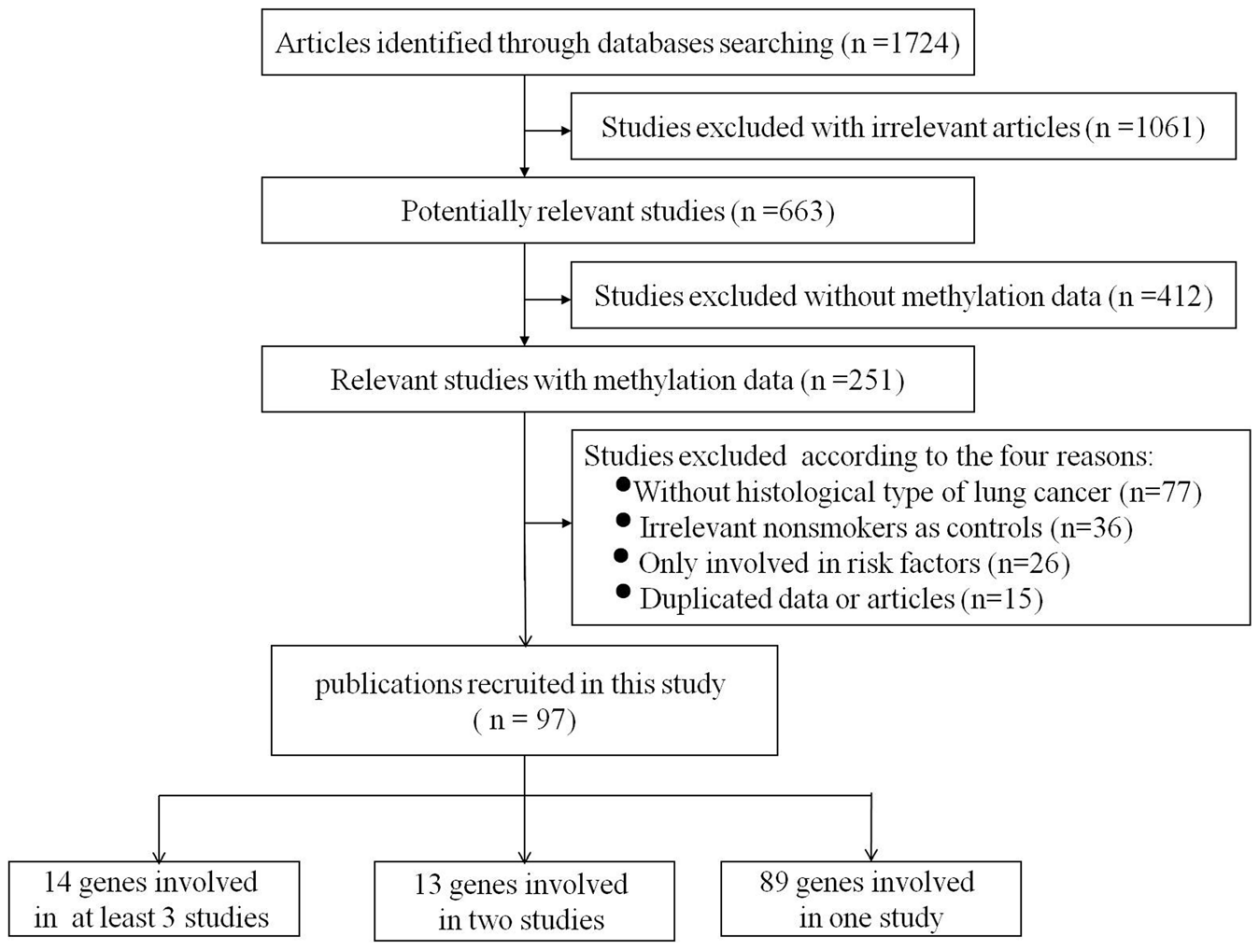
Flow diagram of selecting studies for meta-analysis.

As shown in the Fig. 1, our initial search for the genetic studies of NSCLC retrieved 1724 articles from PubMed, CNKI and Wanfang literature databases. Among the retrieved studies, there were 1061 irrelevant studies, 412 studies without methylation data, 77 studies without the histological types of lung cancer, 15 studies with duplicate data, 26 only involved in risk factors, and 36 studies without smoking status of participants. At last, a total of 97 eligible association studies between smoking and nonsmoking NSCLC patients were included in the current meta-analyses. There were 14 genes involved in at least three studies, 13 genes involved in two studies and 89 genes involved in one study.

Smoking behavior was evaluated for its contribution to the methylation status of 116 genes in NSCLC patients. Among these genes, 14 hypermethylated and 12 hypomethylated genes were shown to be associated with the smoking behavior. Six hypermethylated and eight hypomethylated genes were reported to be associated with smoking in one study (p < 0.05, Dataset 1). In addition, four hypermethylated and one hypomethylated genes were reported to be associated with smoking behavior in two independent studies (p < 0.05, Dataset 1).

**Figure 2:**
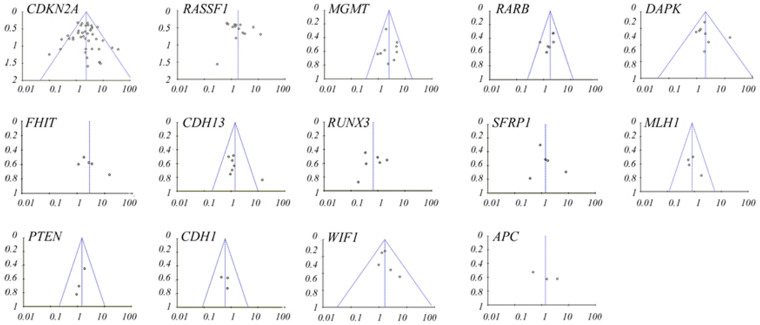
Funnel plots for the relationship between 14 genes and NSCLC in the meta-analyses.

For 14 genes reported in at least three studies (Dataset 1), no evidence of statistical heterogeneity was observed for nine genes, including *CDKN2A* (I^2^ = 38%), *MGMT* (I^2^ = 19%), *CDH13* (I^2^ = 38%), *RARB* (I^2^ = 0%), *DAPK* (I^2^ = 27%), *WIF1* (I^2^ = 0%), *MLH1* (I^2^ = 0%), *PTEN* (I^2^ = 0%) and *CDH1* (I^2^ = 0%). No visual bias was shown in the meta-analyses of the above nine genes (Fig. 2). Our data also demonstrated a significant heterogeneity of the rest five genes that comprised *RASSF1* (I^2^ = 57%), *RUNX3* (I^2^ = 56%), *SFRP1* (I^2^ = 55%), *FHIT* (I^2^ = 52%) and *APC* (I^2^ = 69%). Therefore, random-effect tests were applied for meta-analysis of the above five genes. Their funnel plots were shown in Fig. 2.

As shown in Table 1, meta-analysis of *CDKN2A* gene was involved with 36 studies between 2957 smoking and 1192 nonsmoking NSCLC patients. Our results indicated that *CDKN2A* hypermethylation was significantly associated with smoking risk in NSCLC patients (the overall OR = 2.33, 95% CI = 1.96–2.77, p < 0.00001). Meta-analysis of the association between *RASSF1* hypermethylation and smoking behavior among 1046 smoking NSCLC patients and 441 nonsmoking NSCLC patients indicated a statistical difference (the overall OR = 1.75, 95% CI = 1.15–2.65, p = 0.008). The same consequences were also found in the other five genes including *MGMT* (the overall OR = 2.51, 95% CI = 1.81–3.46, p < 0.00001), *RARB* (the overall OR = 1.77, 95% CI = 1.29–2.42, p = 0.0004), *DAPK* (the overall OR = 2.04, 95% CI = 1.40–2.99, p = 0.0002), *WIF1* (the overall OR = 1.62, 95% CI = 1.04–2.53, p = 0.03) and *FHIT* (the overall OR = 2.18, 95% CI = 1.33–5.95, p = 0.007). Our meta-analyses were unable to find statistical association with smoking for the methylation of the rest 7 genes, including *CDH13*, *RUNX3*, *SFRP1*, *MLH1*, *APC*, *PTEN* and *CDH1*.

**Figure 3:**
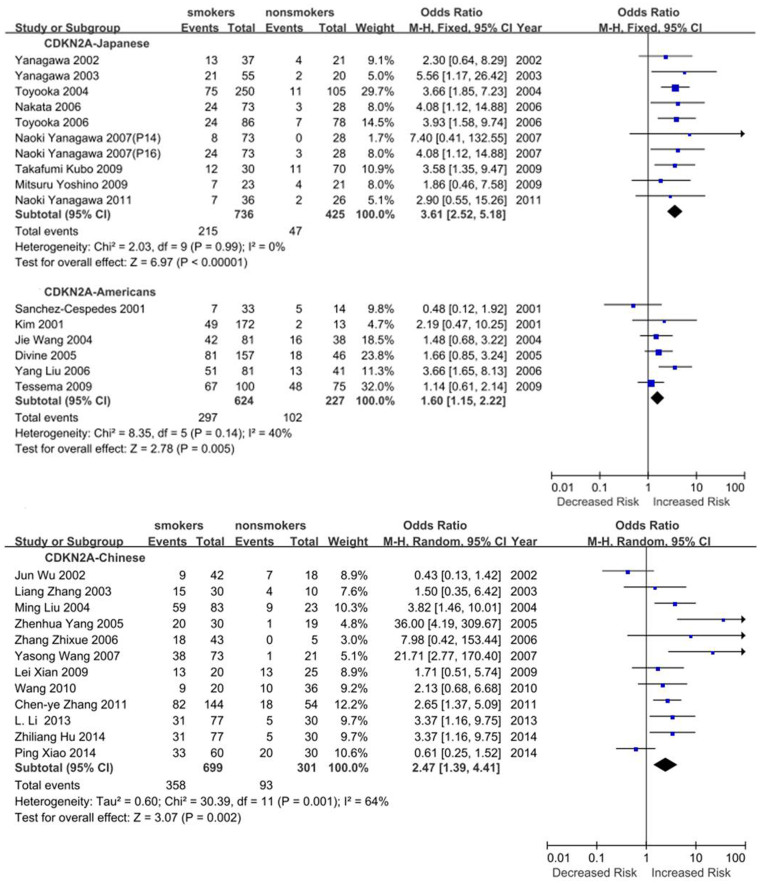
Forest plots of the association studies between *CDKN2A* and NSCLC.

**Figure 4:**
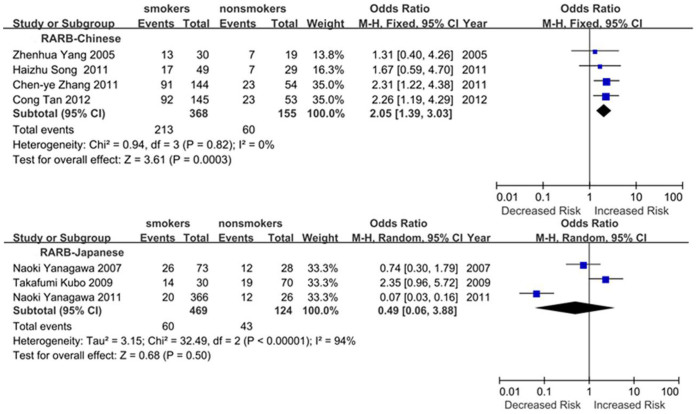
Forest plots of the association studies between *RARB* and NSCLC.

**Figure 5:**
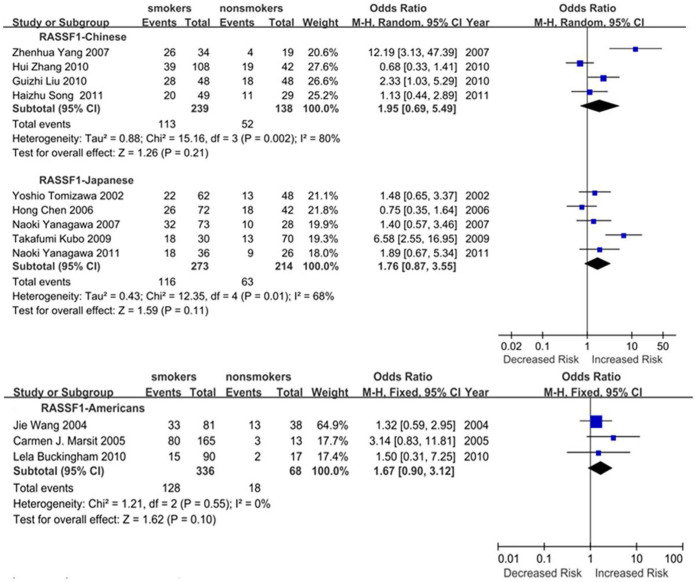
Forest plots of the association studies between *RASSF1* and NSCLC.

**Figure 6:**
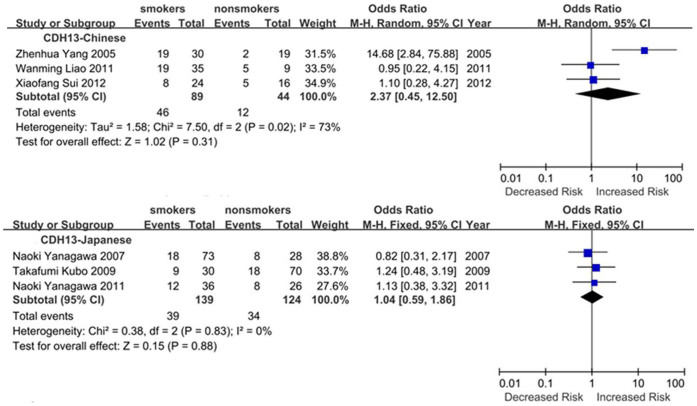
Forest plots of the association studies between *CDH13* and NSCLC.

**Figure 7:**
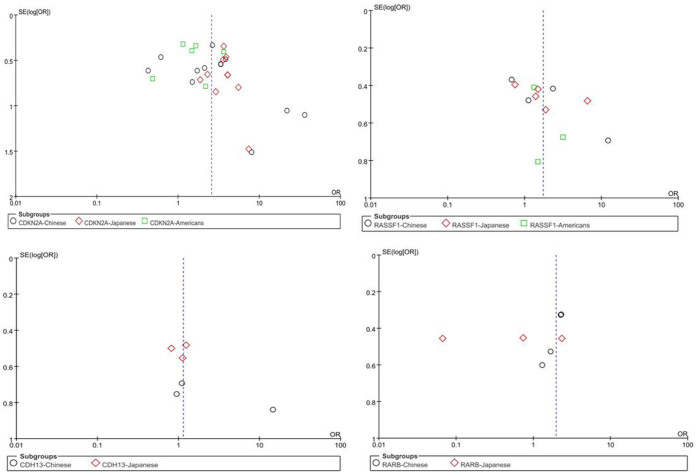
Funnel plots for the relationship between four gene methylation and smoking exposure among NSCLC by region subgroup.

Subgroup meta-analyses by ethnic populations were also performed for *CDKN2A*, *RASSF1*, *CDH13* and *RARB*. As shown in Fig. 3, our results showed that significant association between cigarette smoking and *CDKN2A* hypermethylation existed in Japanese from 10 studies (OR = 3.61, 95% CI = 2.52–5.18), Americans from 6 studies (OR = 1.60, 95% CI = 1.15–2.22) and Chinese from 12 studies (OR = 2.47, 95% CI = 1.39–4.41). In addition, there was a significant ethnic difference in the meta-analysis of *RARB* gene. We found a statistical difference between *RARB* hypermethylation and smoking status in Chinese (OR = 2.05, 95% CI = 1.39–3.03, I^2^ = 0) but not in Japanese (p = 0.50, I^2^ = 94%, Fig. 4). Subgroup meta-analyses of *RASSF1* and *CDH13* were unable to observe any significant results in each ethnic population (Fig. 5 and Fig. 6). The funnel plots of four genes showed there was no publication bias among the above meta-analyses (Fig. 7).

## Discussion

In the present study, a comprehensive overview of genetic association studies was performed for the susceptibility of smoking patients of NSCLC. Our meta-analyses mainly focused on 14 tumor suppressor genes (≥3 studies per gene). Seven NSCLC-associated genes (*CDKN2A*, *RASSF1*, *MGMT*, *RARB*, *DAPK*, *WIF1* and *FHIT*) hypermethylation showed significant evidences in the smoking NSCLC patients compared with nonsmoking NSCLC patients. Subgroup meta-analyses by ethnic populations found that *CDKN2A* hypermethylation was a common risk factor of cigarette smoking in NSCLC patients, however, *RARB* hypermethylation was only found in Chinese.

Smoking is the main cause of lung cancer^14^,^15^, which can be associated with the calcium signaling and the other signaling pathways^16^,^17^,^18^,^19^. Aberrant methylation of gene promoters induced by cigarette carcinogens was shown to be associated with cancer occurrence and tumor suppressor gene inactivation^20^. Cigarette smoking was shown to induce DNMT1 accumulation and subsequent hypermethylation of the tumor suppressor gene that might cause tumorigenesis and poor prognosis in NSCLC patients^21^.

Cyclin-dependent kinase inhibitor 2A (*CDKN2A*) has two alternative transcripts that playing an important role in the p53 and pRB tumor suppressor pathways^22^. Tobacco carcinogens were shown to raise promoter methylation and then down-regulate the expression of *CDKN2A* gene^23^, which might lead to tumorigenesis. Ras-association domain family (*RASSF1*) and O-6-methylguanine DNA methyltransferase (*MGMT*) gene are both involved in lung cancer development in different human lung cell lines^24^,^25^. Cigarette smoking led to the changes in expression and promoter methylation of *MGMT* and *RASSF1*^26^. Retinoic acid receptor beta *(RARB)* encoded a receptor mediating cellular signaling in embryonic morphogenesis, cell growth and differentiation^27^,^28^. Death-associated protein kinase (*DAPK*), a tumor suppressor gene, encodes a novel serine/threonine kinase linked with loss of expression in cancers^29^,^30^. Fragile histidine triad (*FHIT*) gene is located in the most common fragile site that is easy to be rearranged with the exposure in tobacco and other carcinogens, finally causing malignant transformation^31^. WNT inhibitory factor 1 (*WIF1*), as another tumor suppressor gene, has been found to be epigenetically silenced in various cancers including NSCLC^32^. Our results indicated that the hypermethylation of these genes were associated with cigarette smoking in the development of NSCLC.

Our meta-analyses aimed to provide a comprehensive list of aberrantly methylated genes in the smoking NSCLC patients. Compared with the meta-analysis of *CDKN2A* by Zhang et al^33^, our study was involved with a total of 2957 smoking patients and 1192 nonsmoking patients, which provided a much larger sample size than 2037 smoking patients and 765 nonsmoking patients in the study by Zhang et al^33^. In addition, our study was involved with the meta-analysis of 14 genes, among which 10 genes (including *MGMT*, *DAPK*, *FHIT*, *CDH13*, *RARB*, *SFRP1*, *PTEN*, *CDH1*, *WIF1* and *APC*) hadn't been included by previous meta-analysis studies. Moreover, the meta-analysis by Liu et al.^34^ showed that *RASSF1* gene promoter methylation was not associated with smoking behaviour in NSCLC patients. Our meta-analysis of *RASSF1* methylation involved with 14 studies, which was 6 more than that of Liu et al.^34^. The smaller sample size might be the reason for the negative results in the meta-analysis by Liu et al.^34^.

Previous studies found mixed results regarding to racial disparities in NSCLC^35^. Our population-based subgroup meta-analyses showed that significant association of *CDKN2A* hypermethylation existed in Japanese, Chinese and American populations. However, *RARB* hypermethylation was only found in Chinese but not in Japanese.

There were several potential limitations in our study. Firstly, selection bias might exist since publications only in English and Chinese were included in the current meta-analyses, which could affect the results in a certain extent. Secondly, the main ethnicities of the current meta-analyses were Chinese, Japanese and Americans. Studies in other ethnic populations were needed in the near future to explore the contribution of gene methylation to the smoking in NSCLC. Thirdly, the effects of genetic factors on NSCLC risk might be confounded by gender^36^ and age^37^, therefore better design were warranted to avoid these confounding factors in the future. Lastly, our meta-analysis focused on gene with at least three independent studies, and this might prevent those genes reported in two large scale smokers-nonsmokers studies from being included in the current meta-analysis.

In conclusion, we identified significant associations between seven genes (*CDKN2A*, *RASSF1*, *MGMT*, *RARB*, *DAPK*, *WIF1* and *FHIT*) and smoking behavior in non-small cell lung cancer patients. *CDKN2A* hypermethylation was a common risk factor of smoking behavior in NSCLC patients, however, *RARB* hypermethylation was only found as a risk factor of smoking in Chinese but not in other populations.

## Author Contributions

T.H., X.C., S.D. and M.Y. contributed to the conception, design and final approval of the submitted version. Q.H., Z.D., H.M., Y.X., Y.F., H.Y., R.W. and C.Z. contributed to the meta-analysis, interpretation of data and completion of figures and tables. All the authors read and approved the final manuscript.

## Figures and Tables

**Table 1:** Characteristics of 14 genes# among smoking behavior

Gene	Studies (n)	Overall OR (95% CI)^a^	I^2^	*P* value	smoking/non-smoking NSCLC patients (n)
*CDKN2A*	36	2.33 [1.96, 2.77]	38%	<0.00001	2957/1192
*RASSF1*	14	1.75 [1.15, 2.65]	57%	0.008	1046/441
*MGMT*	8	2.51 [1.81, 3.46]	19%	<0.00001	478/339
*RARB*	7	1.77 [1.29, 2.42]	0%	0.0004	507/279
*DAPK*	7	2.04 [1.40, 2.99]	27%	0.0002	427/192
*FHIT*	5	2.81 [1.33, 5.95]	52%	0.007	406/112
*CDH13*	7	1.42 [0.93, 2.18]	38%	0.11	300/184
*RUNX3*	6	0.67 [0.34, 1.34]	56%	0.26	303/156
*SFRP1*	6	1.40 [0.75, 2.61]	55%	0.29	358/190
*MLH1*	4	0.82 [0.46, 1.44]	0%	0.48	225/86
*PTEN*	3	1.47 [0.74, 2.91]	0%	0.27	229/98
*CDH1*	3	0.61 [0.31, 1.21]	0%	0.16	132/61
*WIF1*	5	1.62 [1.04, 2.53]	0%	0.03	346/161
*APC*	3	1.36 [0.41, 4.55]	69%	0.62	89/66

I^2^ stands for heterogeneity;

*P* value stands for significant or insignificant results;

a: Overall OR describes the likelihood of gene methylation and smoking status observed in NSCLC patients;

# means only the genes over 3 studies or equal to 3 studies were displayed, the other genes were listed in Dataset 1.
